# Enabling Fast AI-Driven Inverse Design of a Multifunctional Nanosurface by Parallel Evolution Strategies

**DOI:** 10.3390/nano15010027

**Published:** 2024-12-27

**Authors:** Ashish Chapagain, Dima Abuoliem, In Ho Cho

**Affiliations:** Department of Civil, Construction, and Environmental Engineering, Iowa State University, Ames, IA 50011, USA; cashish@iastate.edu (A.C.); abuoliem@iastate.edu (D.A.)

**Keywords:** capillary force lithography, parallel evolution strategies, light-controlled nanopatterning, AI-driven inverse design, multifunctional nanosurface

## Abstract

Multifunctional nanosurfaces receive growing attention due to their versatile properties. Capillary force lithography (CFL) has emerged as a simple and economical method for fabricating these surfaces. In recent works, the authors proposed to leverage the evolution strategies (ES) to modify nanosurface characteristics with CFL to achieve specific functionalities such as frictional, optical, and bactericidal properties. For artificial intelligence (AI)-driven inverse design, earlier research integrates basic multiphysics principles such as dynamic viscosity, air diffusivity, surface tension, and electric potential with backward deep learning (DL) on the framework of ES. As a successful alternative to reinforcement learning, ES performed well for the AI-driven inverse design. However, the computational limitations of ES pose a critical technical challenge to achieving fast and efficient design. This paper addresses the challenges by proposing a parallel-computing-based ES (named parallel ES). The parallel ES demonstrated the desired speed and scalability, accelerating the AI-driven inverse design of multifunctional nanopatterned surfaces. Detailed parallel ES algorithms and cost models are presented, showing its potential as a promising tool for advancing AI-driven nanomanufacturing.

## 1. Introduction

AI and machine learning (ML) have appeared as transformative techniques in nanostructure design, mainly for solving inverse design problems, which are complex and computationally intensive due to their nature and dimensionality. In recent years, the complexity of nanostructure design has grown significantly, emphasizing the efficiency of implementing AI and ML to accelerate the design process [1].

In advanced technology sectors, nanoscale devices hold significant value due to the increasing demand for compact, multifunctional devices. Nature inspires researchers in every domain; for example, the wings of glasswing butterflies possess antireflection and antifouling properties, making them suitable for optical implants [2]. In a similar stream, cicadae possess antibacterial properties solely due to the interaction between bacteria and the nanostructures on their surface [3], *Spodoptera eridania* (nocturnal moths) have enhanced light perception by minimizing light reflection through matching the refractive index of air with the lens [4], and the unique riblet structure on shark skin helps reduce drag, increasing its efficiency and speed while swimming [5]. Mimicking these natural properties can be helpful in multiple disciplines for achieving different functionalities. Researchers have been able to develop nanoscale devices that have antireflection and antifouling properties, as inspired by the glasswing butterflies [2], and simulations of a marine vessel after modifying its hull design using a riblet structure, as inspired by shark skin, showed a reduction in drag [5]. One of the physical properties that influence the functionality of the surface is the height of the nanostructures and their distribution [6]. The process of creating these surfaces and their structures is known as nanofabrication, and it is essential to produce the nanostructures in a cost-effective manner.

Ref. [7] presents a work showing the potential of ML for discovering the nanomaterial by predicting the cathode in the rechargeable batteries made of zinc, selecting 80 from 130,000 materials with 70 never tested before. The nanotechnology implements ML techniques by using convolutional neural networks (CNNs) to analyze the scanning images of nanosurfaces, getting 95% accurate classification of the nanoparticles. ML shows a high ability to predict the properties of the nanomaterials and can guide the design process and the invention of nanomachines by creating a vast dataset for training the model includes two main key tasks: the automation of tools implemented from quantum data and the simulation of nanodevices without a human in the process [1]. The quantum dataset simulates the nanodevice’s appearance and operations with AI, improving the nanoscale production, such as nanoparticles used for LEDs (perovskites) and catalytic bio-molecules. The investigation of plasmonic nanostructure parameters’ relation between nanodisk geometry and spectra showed that ML can predict cases with less than 5% errors to the truth performance [8]. Ref. [9] used a type of CNNs, specifically a residual network, including various sequential units. Each unit consisted of three layers: a convolution, a batch, and an activation layer. The network analyzes a 2D image of nanostructure data, capturing the shape type, position, orientation, and dimension information.

Ref. [10] investigated the application of ML in designing nanomaterial phases for sustainable development and environmental risk assessment (ERA). The study highlighted different methods, such as regression models and CNNs, to predict the material’s toxicity according to the size, shape, composition, and properties like thermal stability and conductivity. Ref. [11] studied the design of carbon nanotubes (CNTs) as nanomaterials for the lubrication of different types of polymers using an ML model. Their results proved the ability of the model to discover the complex relation between key parameters. Identifying the parameters guarantees accurate predictions of CNT friction and wear behavior, thereby setting a framework for optimizing different friction conditions. Ref. [12] used ML in predicting the antibacterial capacity of nanoparticles by training regression algorithms and diverse validation metrics. Their results showed that 78% of the dependent variables are defined by the model prediction, with key predictive variables including the size of the nanoparticle core, bacterium species, and exposure dose.

ML demonstrated its effectiveness in nanoengineering, especially in the artificial neural networks (ANNs) models. Ref. [13] analyzed the physicochemical of nanoparticles profile with zeta potential predictions by the temperature, pH, and ionic content of dispersions. Their results showed that the zeta value increased by optimizing the manufacturing parameters, reducing the nanoparticle agglomeration, and improving production sustainability as reinforcements. Ref. [14] included in their study the deploying of ML for precise crystallographic predictions at the nanoscale, seeking to enhance the design and experiment process. The model used for prediction was created with a support vector machines (SVM) model optimized with a genetic algorithm (GA) that defines the molecular edge, connectivity, functional correlation, and structure properties.

Conceived in the 1900s with nanoimprint lithography [15,16,17,18,19,20], which is a mechanical process that impresses nanoscale features from a pre-patterned surface (template) to a substrate, several lithography techniques are in use for nanofabrication. Photolithography uses light to transfer a geometric pattern from a photomask to a light-sensitive chemical photoresist [21,22]. Focused ion beam lithography [23] uses beams of ions to mill the material surface with high precision. CFL leverages capillary force using ultraviolet (UV) curable polymers without the need for additional pressure [24,25,26]. The basic working principle of CFL is that when a liquid-phase polymer comes into contact with a polydimethylsiloxane (PDMS) mold, where the liquid’s total free energy is reduced drives the liquid to rise above the capillary [26,27].

**Figure 1:**
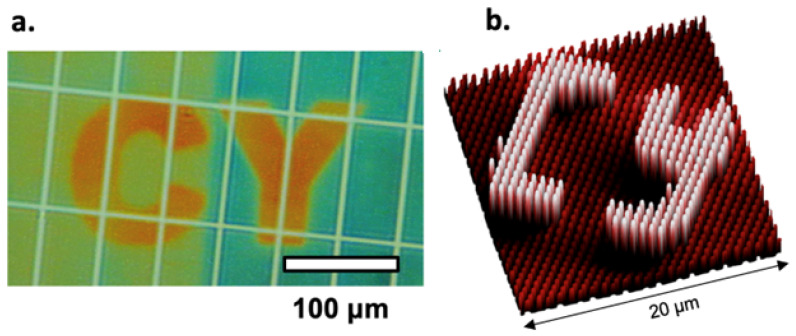
Fabrication and visualization of the “CY” pattern using CFL. (**a**) The fabrication of the “CY” pattern on the nanosurface using CFL, revealing the capability to achieve distinct colors by precisely controlling nanoridge heights to specific dimensions. (**b**) A 3D representation of the “CY” pattern showing the height of nanoridges distribution, where variations in height due to the observed color distinctions. ((**a**,**b**) are cited from [28]).

Among various nanofabrication techniques, CFL offers several advantages. CFL is generally less expensive than many high-resolution techniques, such as focused ion beam lithography, because it does not need specialized equipment or chemical processes [28]. In addition, CFL does not rely on high-energy sources like focused ion beam lithography and photolithography; instead using capillary forces to shape nanostructures, making this technique more simple and energy efficient. The discrepancy between predicted and fabricated nanosurfaces has been addressed by one of our collaborators [28] through precise height modeling of nanostructures using CFL, enabling successful pattern fabrication, such as the letters “CY”. This is achieved with variations in the height of nanoridges, which correlated with distinct colors. Using CFL, the nanoridge module has heights of 1123 ± 16 nm, 965 ± 8 nm, and 848 ± 34 nm, resulting in the colors orange, yellow, and blue, respectively. These structures were achieved using a PDMS mold featuring a height of 1200 nm, width of 1500 nm, and pitch grating of 3000 nm. Figure 1a presents the color changes through height variation in the letters and background, while Figure 1b shows the precise fabrication of the pattern “CY.” The alignment between the model and the experiment results demonstrated the ability to achieve accurate predictions using parallel ES for nanosurface fabrication.

Thus, in this study, nanostructures are fabricated using light-controlled CFL, which involves pre-curing the photopolymer Norland Optical Adhesive (photopolymer–NOA) using UV radiation, modifying its properties, and affecting capillary action. One problem persists in light-controlled CFL—the “forbidden gap”, which is the sudden drop in height attained by nanostructures when a specific threshold UV dose is reached [6].

In recent advances in addressing the problem of the “forbidden gap”, researchers sought to uncover the pseudo-physics governing the nanoscale phenomenon [29]. Using a hybrid intelligence framework, the authors identified the fundamental physics influencing a liquid’s rise in nanoscale. The identified physical properties were air diffusivity, dynamic viscosity, surface tension, and electric potential, vital in controlling nanoscale height modulation during the CFL process. This study enabled the authors to predict and control the nanostructure’s height by overcoming the sudden jump [29]. The limited data could then be enriched using pseudo-physics principles, and the required UV dose could be predicted to attain a specific nanostructure height, thereby allowing more control over the fabrication process. This represents a notable advancement and provides a reliable framework for nanoscale fabrication. Further advances were made to incorporate the pseudo-physics to design a surface with multifunctional surfaces.

In [30], the researchers introduced an algorithm capable of designing multifunctional nanosurfaces with on-demand color, bactericidal, and frictional properties. These properties are quantified using three objective functions—${\mathrm{Obj}}_{\mathrm{ab}}$, ${\mathrm{Obj}}_{f}$, and ${\mathrm{Obj}}_{c}$. Each objective function is normalized to a scale of 0 to 1, with 1 representing an ideal fulfillment of the target property. The cumulative objective function, Obj, combines these three individual objectives, optimizing the nanosurface design to meet all target properties effectively:

(1) $$\mathrm{Obj}=\frac{1}{3}\left({\mathrm{Obj}}_{\mathrm{ab}}+{\mathrm{Obj}}_{f}+{\mathrm{Obj}}_{c}\right)$$

**Figure 2:**
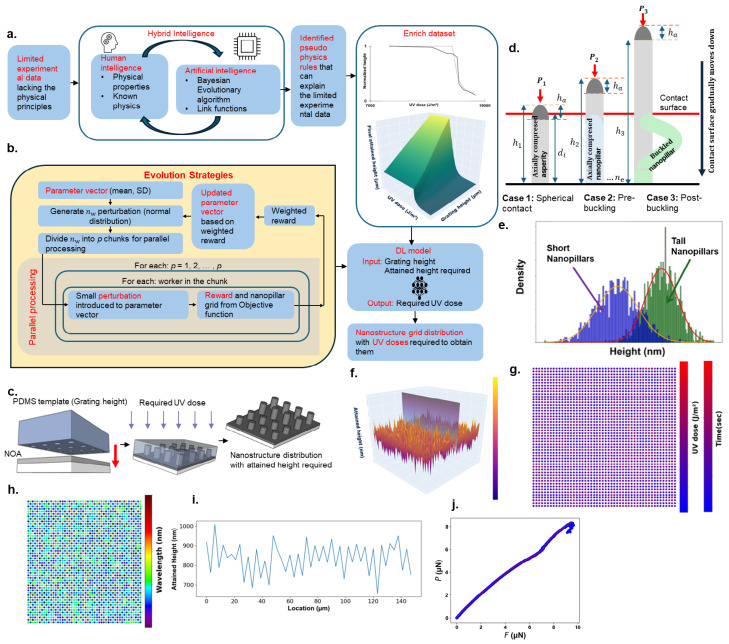
(**a**) The framework illustrates a hybrid intelligence approach, combining humans and AI to address limited experimental data in the physical sciences. Pseudo-physics rules, derived from human insights and machine computation, enhance the dataset with insights on UV dosage requirements. DL models predict the necessary UV dose based on the CFL template pattern and attained heights, while ES optimizes for functional properties, such as friction, color, and antibacterial effectiveness. Using ES and DL in unison, the UV dose required for nanostructure height in a grid distribution can be accurately predicted and fabricated. (**b**) Workflow of ES implemented on multiple processors to leverage parallel processing, distributing tasks across processors for efficient optimization. Each processor operates a segment of worker tasks, collectively refining functional properties for improved performance. (**c**) The process of CFL, where a PDMS template is lowered into photopolymer–NOA, which is then treated with UV dose, and thus, forms a nanopillar array. (**d**) The descent of a contact surface into nanopillars one at a time, along with three cases of deformation: case 1, spherical contact; case 2, pre-buckling; and case 3, post-buckling state. (**e**) The distribution of tall and short pillars impacts bactericidal properties on a surface. The solid red line depicts the distribution of tall nanopillars, while the solid yellow line illustrates the distribution of short nanopillars. The green bars indicate randomly selected heights from the tall nanopillar distribution, and the blue bars show randomly selected heights from the short nanopillar distribution. (**f**) A 50 × 50 grid of nanopillar arrays representing a 150 µm × 150 µm surface area with multifunctional properties optimized using parallel version ES. (**g**) UV dose required to generate the surface and the time needed to achieve this dose at an intensity of 150 J/m^2^/s. (**h**) Color of the surface generated by the sequential version for a target wavelength of 500 nm, achieving the desired green hue. (**i**) Cross-sectional height variation along the surface marked in (**f**). (**j**) Normal force (*P*) vs. frictional force (*F*) plot, where the slope of the curve reflects the required friction coefficient (μ), reaching the target value of 2.41.

Thus, a cumulative score of Obj=1 would signify an optimal design, achieving the highest standards across bactericidal, frictional, and color properties. For the purpose of this paper, the lower threshold for the objective function value is taken as 0.96. In particular, the antibacterial objective function, ${\mathrm{Obj}}_{\mathrm{ab}}$, quantifies the bactericidal effectiveness of the surface: ${\mathrm{Obj}}_{\mathrm{ab}}=f\left(1-\frac{|\delta_{t}-\delta_{p}|}{\delta_{t}}\right)$, where $\delta_{t}$ is the target difference between the average height of short and tall nanostructures and $\delta_{p}$ is the predicted difference between the heights. The function f(x)=exp(x−1) normalizes this difference, allowing ${\mathrm{Obj}}_{\mathrm{ab}}$ to range from 0 to 1, with 1 indicating perfect bactericidal effectiveness. The frictional objective function, ${\mathrm{Obj}}_{f}$, evaluates the surface’s frictional property: ${\mathrm{Obj}}_{f}=f\left(1-\frac{|\mu_{t}-\mu_{p}|}{\mu_{t}}\right)$, where $\mu_{t}$ is the target friction coefficient and $\mu_{p}$ is the predicted value, and the objective function is normalized using f(x)=exp(x−1). ${\mathrm{Obj}}_{f}$ also ranges from 0 to 1, with 1 indicating a perfect match to the target frictional property. The color reproduction objective function, ${\mathrm{Obj}}_{c}$, measures the accuracy of color matching: ${\mathrm{Obj}}_{c}=g\left(D_{\mathrm{KL}}\left(f_{p}\|f_{t}\right)\right)$, where $D_{\mathrm{KL}}\left(f_{p}\|f_{t}\right)$ is the Kullback–Leibler (KL) divergence between the predicted $\left(f_{p}\right)$ and target $\left(f_{t}\right)$ height distributions, normalized with $g\left(x\right)=\frac{2}{1+\exp(x/20)}$, to map the value between 0 and 1. Here, ${\mathrm{Obj}}_{c}=1$ represents perfect color reproduction. One of the most dominant tasks in computing the objective function is finding the friction coefficient, which directly affects the aim function for friction [30]. The nanopillars are designed with a top hemisphere and cylindrical pillar (see Figure 2d). To understand the relationship between the normal force (*P*) and the frictional force (*F*), a three-stage approach was adopted. In the first stage, contact occurs at the hemispherical top, and a small deformation is observed (see Figure 2d, case 1). Young’s modulus (*E*) and Poisson’s ratio (ν) of PDMS were used to define the composite elastic modulus as $E^{*}=\frac{E}{1-\nu^{2}}$. Under shear, the contact area of the hemisphere changes from an initial area ($a_{0}$) to a final area ($a_{f}$), where $B=\frac{a_{f}}{a_{0}}$ represents the extent of this change. At the microscopic level, for each nanopillar’s hemisphere, the initial contact area for the *i*-th microcontact is defined as $a_{0,i}=\pi R\left(h_{i}-d\right)$ if $h_{i}\geq d$ and $a_{0,i}=0$ if $h_{i}<d$. This case is identified based on the condition $h_{i}-d<h_{a}$, where $h_{a}$ is the height of the hemispherical top. Moreover, *d* represents the distance between the contact surface and the base, $h_{i}$ is the height of nanostructures, and *R* is the hemisphere is radius of curvature. The final contact area for each hemisphere is $a_{f,i}$, where $f_{i}$ indicates the frictional force acting on the hemispherical top. The frictional force (*F*) scales with the contact area (*A*) and is expressed as $f=sa_{f}$, where *s* is the frictional strength at the PDMS–glass interface.

On a macroscopic level, for *N* asperities, the total initial and final contact areas are $A_{0}=\sum_{i=1}^{N}a_{0,i}$ and $A_{f}=\sum_{i=1}^{N}a_{f,i}$, respectively, with each $a_{f,i}=B\times a_{0,i}$. Using Hertz’s model, the normal force *P* is calculated as:

(2) $$P_{i}=\left(\frac{4E^{*}}{3}R^{1/2}{\left(h_{i}-d\right)}^{3/2}\right),$$

and the frictional force as:

(3) $$F_{i}=Bs\pi R\left(h_{i}-d\right).$$

This stage remains valid as long as contact is limited to the hemispherical top of the nanopillars.

The second stage begins when the contact surface reaches the cylindrical pillars. At this point, the cylindrical part of each nanopillar undergoes axial compression. For each cylindrical portion, the axial force $P_{i}$ is calculated using Hooke’s law as:

(4) $$P_{i}=\left(\frac{\pi r^{2}E^{*}}{h_{i}}\left(h_{i}-d\right)\right),\;\forall i\in \left[1,N\right]$$

with lateral resistance for each pillar determined by:

(5) $$F_{i}=s\pi r^{2}.$$

The final third stage occurs if the normal force $P_{i}$ causes the nanopillars to buckle. This stage requires evaluating the potential for buckling through Euler’s buckling formula for a circular section, which assumes fixed support at the nanopillar base and hinged support at the top, which can be identified when $h_{i}-d<h_{a}$ and $P_{i}>P_{\mathrm{cr}}^{i}$:

(6) $$P_{\mathrm{cr}}^{i}=\frac{\pi^{2}E^{*}I}{{\left(0.7h_{i}\right)}^{2}}$$

where *I* is the second-moment area of the circular nanopillars. In the event of buckling, the nanopillars are considered redundant in terms of frictional force, making both $P_{i}$ and $F_{i}$ zero for buckled pillars.

Ultimately, the total normal force *P* and frictional force *F* across a surface with *N* nanopillars are calculated as the summation of individual forces from each nanopillar, after checking all three cases for each nanopillar in the nanostructure array:

(7) $$P=\sum_{i=1}^{N}P_{i}\;\mathrm{and}\;F=\sum_{i=1}^{N}F_{i}.$$

The authors proposed a computational model that facilitated the rapid fabrication of nanostructures. In this study, the researchers address the scalability challenges of the sequential version of the ES algorithm by implementing a parallel version of the ES algorithm based on the work of [31]. While the previous work developed a serial version of an AI-driven multifunctional nanopattern using ES, this process successfully optimized multifunctional nanosurfaces but faced limitations, especially with large-scale or complex designs. However, the current work handled these limitations by introducing a parallel version of the ES algorithm. Parallel computing has been essential for solving large-scale and complex optimization problems in various fields. Hierarchical grouping strategies can improve multiscale analytical performance in earlier parallel computing studies. Researchers have elevated computational efficiency to near-superlinear speeds in some cases by controlling the distributing workloads across symmetric multiprocessor (SMP) clusters and minimizing inter-processor communication [32,33,34]. By efficiently partitioning and subdividing the problem space, these hierarchical grouping techniques enable more effective optimization of complex, nonlinear systems by guaranteeing that each processor functions with minimal communication overhead. This strategy is essential to our study, as the parallel version of the ES algorithm gains a great deal from effective communication techniques, especially when scaled to a larger number of workers.

By distributing the computational load across multiple processors, the parallel ES algorithm provides a significant advantage in scalability and reduces the overall runtime of the optimization process. By transitioning to a parallel version of ES enabled the authors to explore a more extensive solution space using multiple cores in high-performance computing (HPC) clusters. This transition was believed to reduce the execution time for each loop and provide a robust exploration of search space. Figure 2a shows the workflow for implementing Hybrid Intelligence (HI) to generate a multifunctional nanosurface, adapted from [30]. AI-driven methods such as Bayesian Evolutionary algorithms are combined with human knowledge in physical properties to enhance the limited experimental data. Objective and probability density functions are developed to quantify and evaluate the expected properties, and a DL model estimates the needed UV dosages for specific nanostructure heights. The parallel version of the ES algorithm (Figure 2b) distributes worker tasks across multiple parallel units, where each worker independently evaluates and optimizes its assigned task. This method significantly accelerates the optimization process by aggregating each worker’s results to enhance the overall reward efficiently. In addition, Figure 2c demonstrates the CFL fabrication procedure, where a template is lowered to come into contact with a photopolymer–NOA, after which the required UV dose is applied, causing the photopolymer–NOA to rise into the template, thus forming the nanopillar array. Figure 2d depicts the concept for computing friction and normal force, which ultimately determine the friction coefficient (μ). The computation involves three cases, which check the contact section (hemisphere or cylindrical portion) and buckling effect. Figure 2e illustrates the distribution of short and tall nanopillars formed using the parallel version of the ES algorithm employed in the paper, which forms the 3D surface depicted in Figure 2f, with a cross-sectional surface shown in Figure 2i. Figure 2g provides the required UV dose needed to form the surface and the color generated by the surface is shown in Figure 2h. Finally, the *P* vs. *N* diagram, which represents the frictional performance of the surface, is shown in Figure 2j.

## 2. Materials and Methods

The materials and methods section outlines the frameworks and methodologies used in this study to achieve the research objectives. By describing the sequential and parallel versions of the ES algorithm, the implementation of the parallel algorithm by Open_MPI, and the setup of validating model predictions. Furthermore, the computational environment parameters and procedures for performance evaluation are outlined, including the cost analysis of achieving consistency between the sequential and parallel methods.

### 2.1. Sequential Version of ES

A variation of the ES algorithms, adapted from [31], was used for our study, which is presented in Table 1. ES optimizes the distribution of short and tall nanostructures on a multifunctional surface. This distribution is described in terms of the height of short nanostructures and the standard deviation of short and tall nanostructures.

In ES, a parameter vector θ is initialized with three values: $\theta_{1}$, representing the initial guess for the mean height of short nanostructures (nm); $\theta_{2}$, representing the standard deviation of the height of short nanostructures (nm); and $\theta_{3}$, representing the standard deviation of tall nanostructures (nm). For each iteration, a perturbed version of this vector, $\theta_{p}$, is calculated by introducing a slight change in each parameter using each row ($\epsilon_{w}$) of the matrix E. The matrix E has dimensions $n_{w}\times 3$, where $n_{w}$ represents the number of workers (or variations explored) in ES. This approach enables exploration of the parameter space through slight variations in each $\theta_{i}$, based on a noise standard deviation (σ), where σ∈R.

The objective function, Obj(), integrates three independent objectives—the loss functions for color, friction, and bactericidal effect—using $\theta_{p}$ as input and returning the reward *r* and a matrix G. The matrix G contains $n_{e}$ nanostructure heights arranged in a square with dimensions $\sqrt{n_{e}}\times \sqrt{n_{e}}$, where $n_{e}$ is the number of nanostructures present in the surface. The performance of evolution strategies is evaluated through the weighted reward w, which is calculated as $E^{T}\cdot r$, where r is a vector of rewards from each worker. Additionally, α, where α ∈R, represents the learning rate used in the algorithm, which controls the convergence speed of ES.

After running the ES algorithm for *n* iterations, the output for multifunctional nanosurface optimization includes the optimized parameter vector ($\theta_{p}$), a list of average rewards from each iteration ($r_{a}$), and the nanopillar grid matrix (G) that exhibits the desired properties.

### 2.2. Parallel Version of ES Using Open_MPI

ES supports parallelization for parameter exploration and reward aggregation. Each worker task, discussed in the previous section, which is performed sequentially, can be parallelized and distributed among multiple processors. The major parameters of ES, α and σ, remain the same in this version. The parallelized version of ES is presented in Table 2. In the algorithm, Message Passing Interface (MPI) variables such as comm, rank, and size manage the parallel processing setup by facilitating inter-processor communication. Each processor handles a subset, $C_{k}$, of the matrix E. The variables $r_{w}$ and $G_{w}$ represent the reward and the nanostructure grid matrix generated from each processor. Similarly, $r_{w}$ aggregates all $r_{w}$ values. The aggregated variable $r_{w}$ from all processors is gathered into a single vector, $r_{f}$.

After running the algorithm for *n* iterations and accumulating the rewards from each processor, the same approach as in the sequential version is used to compute the weighted reward and update the value of θ.

According to [35], the standard deviations in the distribution of short and tall nanostructures in dragonfly wings exhibiting bactericidal properties are 67 nm and 62 nm, respectively. Minor standard deviations can result in a bichromatic surface, as tall and short nanopillar heights can lead to distinct colors. In contrast, a high standard deviation value can negatively affect convergence time. Therefore, a good practice during the execution of the algorithm is to set these two values to 60 nm.

Each processor handles a subset, $C_{k}$, of the matrix E. The variables $r_{w}$ and $G_{w}$ represent the reward and the nanostructure grid matrix generated from each processor. Similarly, $r_{w}$ aggregates all $r_{w}$ values. The aggregated variable $r_{w}$ from all processors is gathered into a single vector, $r_{f}$. After running it for *n* iterations and accumulating the rewards from each processor, the same approach as in the sequential version is used to compute the weighted reward and update the value of θ. According to [35], the standard deviation in the distribution of short and tall nanostructures in dragonfly wings exhibiting bactericidal properties are 67 nm and 62 nm. Minor standard deviation can result in a bichromatic surface, as tall and short nanopillar heights can result in distinct colors. In contrast, a high standard deviation value can have a detrimental effect on the convergence time. Therefore, a good practice used during running the algorithm is to set these two values to 60 nm.

### 2.3. Cost Model and Performance Analysis of the Parallel Algorithm Using Open_MPI

The total running time $T_{\mathrm{total}}$ of the parallel version of the ES involves both computation and communication costs [32]. The current work uses the previous procedure to derive Equations (8)–(12) to evaluate and estimate the implemented parallel strategy performance in this program. The computation cost is primarily determined by the number of workers ($n_{w}$), the number of elements each worker processes ($n_{e}$), and the number of processors (*p*). Additionally, each computation incurs a cost per operation per element. The objective function has a complexity of $n_{e}^{2}+n_{e}\times \log\left(n_{e}\right)$. The objective function is repeated $n_{w}$ times. Therefore, the total computation cost per processor can be expressed as:

(8) $$\mathrm{Total}\;\mathrm{computation}\;\mathrm{cost}=\left[\frac{n_{w}\times \left(n_{e}^{2}+n_{e}\times \log\left(n_{e}\right)\right)}{p}\right]\times \alpha$$

Next, the total number of transferred elements, *N*, comprises only the broadcasted and returned elements. The total number of broadcasted elements is $\frac{n_{w}\times n_{e}}{p}$. Similarly, the total number of returned elements is $\frac{n_{w}\times n_{e}}{p}$. Adding the broadcasted and returned elements together, the total number of transferred elements is:

$$N=\frac{n_{w}\times n_{e}}{p}$$

The total communication cost depends on the communication startup cost *L*, the transfer cost per element β(p), and the total number of transferred elements *N*. The total communication cost is given by:

$$\mathrm{Total}\;\mathrm{communication}\;\mathrm{cost}=p\times \left(L+\beta (p)\times N\right)$$

The transfer cost per element, β(p), is calculated as $\frac{1}{\zeta }\beta_{s}+\left(1-\frac{1}{\zeta }\right)\beta_{d}$, where $\beta_{s}$ represents the intranodal transfer cost, $\beta_{d}$ represents the internodal transfer cost, and ζ is the number of groups of 36 processors, determined by ζ=floor(p/36) if mod(p,36)=0 and ζ=floor(p/36)+1 otherwise. Substituting β(p) and *N* into the total communication cost expression gives:

(9) $$\mathrm{Total}\;\mathrm{communication}\;\mathrm{cost}=p\times \left(L+\left(\frac{1}{\zeta }\beta_{s}+\left(1-\frac{1}{\zeta }\right)\beta_{d}\right)\times \frac{n_{w}\times n_{e}}{p}\right)$$

Expanding this expression yields:

$$\mathrm{Total}\;\mathrm{communication}\;\mathrm{cost}=p\times L+\frac{n_{w}\times n_{e}}{\zeta }\beta_{s}+n_{w}\times n_{e}\beta_{d}-\frac{n_{w}\times n_{e}}{\zeta }\beta_{d}$$

The total running time $T_{\mathrm{total}}$ per iteration is then the sum of the total computation cost and the total communication cost. Combining the terms gives:

$$\begin{array}{cc} T_{\mathrm{total}}= & \left[\frac{n_{w}\times \left(n_{e}^{2}+n_{e}\times \log\left(n_{e}\right)\right)}{p}\right]\times \alpha +p\times L+\frac{n_{w}\times n_{e}}{\zeta }\beta_{s}+n_{w}\times n_{e}\beta_{d}-\frac{n_{w}\times n_{e}}{\zeta }\beta_{d} \end{array}$$

Reorganizing, we get $T_{\mathrm{total}}$ is:

(10) $$\begin{array}{cc} T_{\mathrm{total}}= & \;\frac{n_{w}\times n_{e}^{2}}{p}\times \alpha +\frac{n_{w}\times n_{e}\times \log\left(n_{e}\right)}{p}\times \alpha +p\times L+\frac{n_{w}\times n_{e}}{\zeta }\beta_{s}+n_{w}\times n_{e}\beta_{d}-\frac{n_{w}\times n_{e}}{\zeta }\beta_{d} \end{array}$$

### 2.4. Optimizing Number of Processors

To find the value of *p* that minimizes the total time $T_{\mathrm{total}}$, Equation (10) was first differentiated with respect to *p*, the derivative was then set to zero, and finally, the resulting equation was solved for *p*:

(11) $$\frac{dT_{\mathrm{total}}}{dp}=-\frac{n_{w}\times n_{e}^{2}\times \alpha }{p^{2}}-\frac{n_{w}\times n_{e}\times \log\left(n_{e}\right)\times \alpha }{p^{2}}+L=0$$

which yields:

(12) $$p=\sqrt{\frac{n_{w}\cdot \alpha \left(n_{e}^{2}+n_{e}\cdot \log\left(n_{e}\right)\right)}{L}}$$

## 3. Results

### 3.1. Comparison Between Surfaces Generated

Using both sequential and parallel versions, the authors sought to create a bactericidal surface with a color corresponding to a wavelength of 500 nm (greenish) and a friction coefficient (μ) of 2.41. The parallel algorithm employed 1000 workers spread across 1000 processors to boost processing performance, while the sequential algorithm employed 1000 workers in a single processor. Both the versions of the ES algorithm were able to optimize a surface of size 150 (μm) × 150 (μm), featuring a nanograting array of size 50 × 50 (2500 nanostructures), where 50 represents the number of grating elements along each dimension in the array.

**Figure 3:**
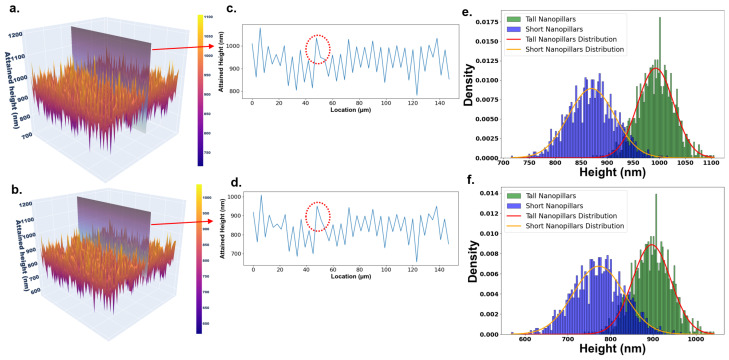
(**a**) A 3D view of the surface generated by the sequential version, representing a 50 × 50 grid of nanopillar arrays over a 150 µm × 150 µm surface area with multifunctional properties. (**b**) A 3D view of the surface generated by the parallel version, also achieving the target wavelength and desired green hue, with a similar 50 × 50 grid of nanopillar arrays for multifunctionality. (**c**) Cross-sectional height variation for the sequential version, showing the nanostructure heights along the section shown in (**a**). (**d**) Cross-sectional height variation for the parallel version, corresponding to the section shown in (**b**). (**e**) Distribution of tall and short nanostructures alongside randomly generated heights following the same distribution for the sequential version. (**f**) Distribution of tall and short nanostructures alongside randomly generated heights for the parallel version, mirroring the distribution in (**e**).

The 3D surfaces generated by the sequential and parallel ES versions are shown in Figure 3a,b, respectively. The study objective is to confirm that the parallel method replicates the design of the sequential method but is not identical by improving computing efficiency. These surfaces exhibit a structured array of alternating tall and short nanopillars arranged in a grid. The cross-sectional height variations of each surface are depicted in Figure 3c,d, emphasizing the uniformity achieved in both versions. While the data seem visually similar, subtle differences are obvious, as underscored by the red circles (Figure 3c,d). Notably, the root mean square error (RMSE) between both versions is 98.04 nm, emphasizing the consistency between the two methods. The differences in height are rooted in the optimization process, with the parallel version converging faster and reaching the target design. The distribution of these nanostructures is further detailed in Figure 3e,f, showing that both versions successfully adhere to the intended pattern, ensuring the design functionalities.

**Figure 4:**
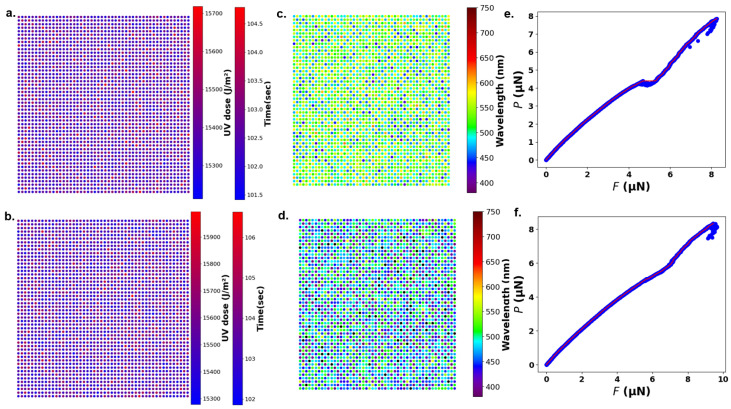
(**a**) UV dose required to achieve the surface shown in Figure 3a for the sequential algorithm, with exposure time at an intensity of 150 J/m^2^/s indicated by a secondary color bar. (**b**) UV dose required for the parallel algorithm to achieve the surface shown in Figure 3b, with corresponding exposure times. (**c**) Color of the surface generated by the sequential version. (**d**) Color of the surface generated by the parallel version. (**e**) Normal force (*P*) vs. frictional force (*F*) plot for the sequential version, where the slope represents the friction coefficient (μ), reaching the target value. (**f**) Normal force (*P*) vs. frictional force (*F*) plot for the parallel version, also achieving the target friction coefficient.

Additionally, the UV dose required to produce each surface in the sequential and parallel versions are illustrated in Figure 4a,b, respectively. This UV dose calculation, based on the grating (template) and individual nanostructure heights as detailed in Figure 2a, was achieved using a DL program as explained in [30]. The surface generated using sequential and parallel ES versions exhibits a greenish color corresponding to a 500 nm wavelength, emphasizing the algorithm’s ability to meet the required surface color and frictional properties. Figure 4c,d further shows the color of the surface formed by the sequential and parallel versions, respectively. Additionally, Figure 4e,f illustrate the normal force (*P*) vs. frictional force (*F*) diagram for the sequential and parallel versions, where the slope of each curve gives the friction coefficient (μ).

Finally, the results demonstrate that both sequential and parallel versions of the ES algorithm yield very similar performance in terms of the surface they form without compromising accuracy or surface quality.

### 3.2. Comparison Between Accuracy and Runtime

**Figure 5:**
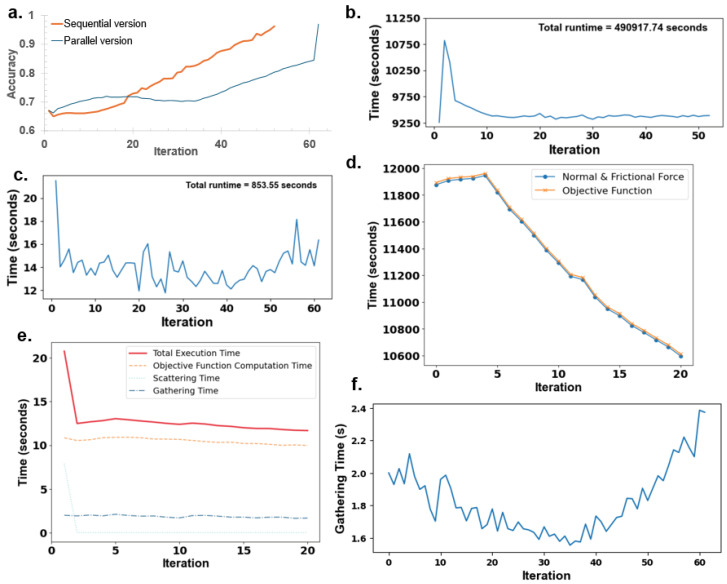
(**a**) Convergence curves of the sequential and parallel versions, demonstrating convergence at 52 and 62 iterations, respectively. While the sequential version converges in fewer iterations, the reduced runtime of the parallel version highlights the efficiency of parallelization in optimizing the multifunctional surface. (**b**) Iteration-wise time taken by the sequential version, revealing substantial computational time per iteration, resulting in a total execution time of 490,917.74 s (approximately 5.6 days). (**c**) Iteration-wise time taken by the parallel version, showing a significant reduction in computational time per iteration, with a total execution time of only 853.55 s, even though more iterations were performed compared to the sequential version. (**d**) Time required per iteration for a separate simulation to investigate spikes using the same parameters. Rerunning the sequential code with these parameters showed iteration times closely match the duration needed for objective function computations, where the most time-consuming part involves normal and frictional force calculations. (**e**) Analysis of time spikes in the parallel version, where total execution time per iteration includes three main tasks: objective function computation (focused on normal and frictional forces), data scattering across processors with *comm.scatter*, and data gathering from all processors to the master processor with *comm.gather*. (**f**) The data gathering phase contributes to the zigzag pattern observed in (**c**).

Figure 5a compares the convergence curves of the sequential and parallel ES versions. Although the sequential version converges in fewer iterations, this advantage becomes redundant as the parallel version’s reduced overall runtime significantly outweighs the sequential version’s faster convergence, thereby underscoring the efficiency of parallelization in optimizing the multifunctional surface. Figure 5b,c compare the time required per iteration. Specifically, Figure 5a shows that the sequential algorithm converged with fewer iterations (52 vs. 62). Figure 5b illustrates the total execution time for the sequential version—490,917 s (5.6 days), compared to the parallel version’s 853.55 s Figure 5c, underscoring the efficiency gained through parallelization. A noticeable initial spike in the time plot shown in Figure 5b corresponds to the objective function computations, which are especially time-intensive in the sequential version, as seen in Figure 5d. This figure shows that the time required for the objective function computation per worker per iteration is nearly equal to the time required for the friction coefficient computation. The objective function consists primarily of the coefficient of friction computation, which directly affects the frictional objective function. This time variation is particularly significant in the sequential version, as shown by the spike in Figure 5b. With 1000 workers on a single processor, the total execution time across all iterations for the frictional and normal force computations ranges from 10,600 to 12,000 s, as seen in Figure 5d, translating to an average of 10.6 to 12 s per iteration per worker. This average per-iteration timing aligns with the parallel version’s per-iteration time for objective function computation, as shown in Figure 5e. Figure 5e includes the itemized time required for all dominant time-consuming tasks in the parallel version.

It is worth noting that Figure 5d,e represent a separate analysis focused solely on the initial portion of the program, covering only the first 20 iterations. Figure 5d shows the time required for computing the objective function, as well as the normal and frictional forces, which nearly coincide with each other, depicting that the objective function computation time is taken mainly by normal and frictional force computation. Figure 5e shows the total execution time taken by the parallel version, which depends upon the time taken by the objective function (normal and frictional force computation, dominantly), scattering time, and the gathering time. Figure 5f illustrates the gathering time, representing the time the master processor requires to collect data from each slave processor per iteration. This gathering time shows a distinct zigzag pattern with spikes, indicating fluctuations in data transfer times throughout the computation process.

### 3.3. Parallel Program Performance

**Figure 6:**
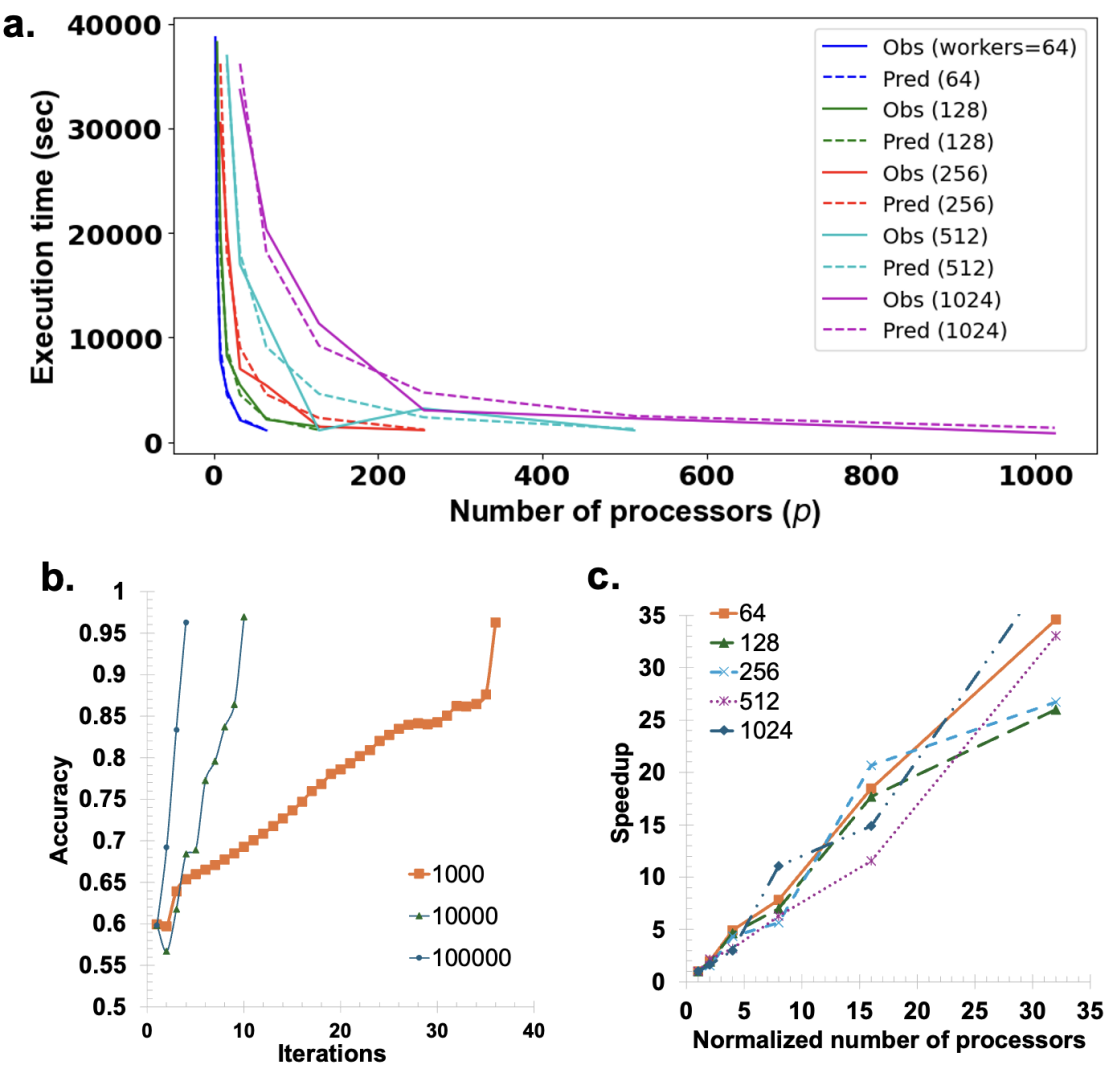
(**a**) Comparison between the predicted execution time for 100 iterations, calculated using Equation (10) with parameters α=0.00018, L=0.0022, $\beta_{s}=1\times 10^{-9}$, and $\beta_{d}=9.46\times 10^{-5}$, and the actual observed total time taken by the program. Results are shown for different processor configurations (64, 128, 256, 512, and 1024 processors), illustrating the algorithm’s scaling performance. (**b**) Convergence behavior of the ES algorithm with a fixed number of processors (p=1000) and varying numbers of workers (1000, 10,000, and 100,000). Convergence occurred at the 33rd iteration for 1000 workers, the 21st iteration for 10,000 workers, and the 8th iteration for 100,000 workers, demonstrating a significant improvement in convergence rate as the number of workers increases. (**c**) Linear speedup achieved using Open_MPI in Python with the parallel algorithm, as detailed in Table 2, showcasing the efficiency gains in processing time due to parallelization using 64, 128, 256, 512, and 1024 workers.

Figure 6a presents a comparison between the predicted execution time for 100 iterations and the actual observed total time across configurations of 64, 128, 256, 512, and 1024 processors. The close alignment between the predicted and actual time graphs for each worker configuration highlights the accuracy of the prediction model, calculated using Equation (10). Figure 6b demonstrates the convergence rate improvements achieved with increasing numbers of workers: configurations with 1000, 10,000, and 100,000 workers converge at 36, 10, and 4 iterations, respectively, showing that higher worker counts lead to significantly faster convergence. Finally, Figure 6c illustrates the linear speedup obtained with the parallel ES using Open_MPI. Here, the y-axis represents speedup, while the x-axis shows the normalized number of processors. For worker counts ranging from 64 to 1024, the graph closely follows the line x=y, indicating a nearly ideal linear speedup.

## 4. Discussion

The results from both the sequential and parallel versions ES algorithm indicate that they perform equivalently in optimizing surface characteristics to achieve the desired color and frictional and bactericidal properties. Both versions successfully generated a surface with a greenish color corresponding to a 500 nm wavelength and a target friction coefficient (μ) of 2.41. Despite the difference in processing configurations with the parallel version utilizing 1000 workers across 1000 processors and the sequential version employing 1000 workers on a single processor, the final surfaces produced were consistent in quality and functional attributes. The structural characteristics of the surfaces generated by each version, including height distribution and nanostructure arrangement, align closely. As shown in Figure 3a–f, both versions formed a uniform grid of 2500 nanopillars within a 150 μm × 150 μm area, arranged in a 50 × 50 matrix of alternating tall and short pillars. The UV dose requirements (Figure 4a,b), the resulting color (Figure 4c,d), and the frictional properties (Figure 4e,f) were also consistent between versions, meeting the desired 500 nm wavelength and friction coefficient (μ) of 2.41. The frictional performance, illustrated by the *P* vs. *F* diagrams in Figure 4e,f, confirms the achievement of the targeted friction coefficient in both cases. These results show that the parallel ES version achieves very similar performance to the sequential version in terms of surface formation, validating it as a scalable and effective solution without compromising quality.

To discuss the implications of Figure 5, we compare the convergence patterns of the sequential and parallel versions. Figure 5a highlights that the sequential version converges in fewer iterations than the parallel version. This quicker convergence, however, is offset by the significant time cost difference illustrated in Figure 5b,c: the sequential version’s total runtime far exceeds that of the parallel version. The parallel implementation’s ability to perform simultaneous computations allows it to complete all iterations in just 853.55 s, a stark contrast to the sequential version’s 490,917 s (or 5.6 days). The initial spike seen in Figure 5c can be attributed to scattering time, as explained in Figure 5e. This scattering overhead results from the initial data distribution to processors, introducing a time cost early in the process. In Figure 5d, we observe that the total runtime depends directly upon the time required for the objective function computation, which is comparable to the time for calculating the normal and frictional forces. These force calculations depend on the nanostructure design (Figure 2d), modeled with hemispherical tops and cylindrical pillars, and its distribution within the grid. The complexity of these friction calculations arises from simulating a gradual descent of the contact surface after sorting nanopillar heights in descending order. Each pillar’s height, $h_{i}$, and the distance between the base and contact surface, $d_{i}$, influence the initial contact area. The deformation initially occurs at the hemispherical tops (as seen in Figure 2d, case 1), where it depends on the composite elastic modulus $E^{*}$, derived from Young’s modulus *E* and Poisson’s ratio ν. In this initial contact phase, the normal and frictional forces *P* and *F* are computed using hemispherical contact formulas (Equations (2) and (3)). However, additional calculations become necessary if the contact extends into the cylindrical portion of the nanopillars (as in Figure 2d, case 2). *P* and *F* in this case are computed using Equations (4) and (5).

If the normal force *P* reaches a critical threshold, pillars may buckle (as in Figure 2d, case 3), rendering them ineffective in contributing to friction. Buckling is evaluated through Euler’s buckling formula (Equation (6)), leading the algorithm to set both $P_{i}$ and $F_{i}$ to zero for buckled pillars. These force calculations are especially sensitive to the height distribution on the grid, which changes randomly with each perturbation and introduces runtime variability. The three cases are determined by the decent of the contact surface and heights of nanopillars, which in turn depends upon the probabilistic approach leading to a spike seen in Figure 5b.

In the parallel version, these calculations still occur, but their impact on runtime is minimized as the load is distributed across processors. However, these computations are now dominated by communication and synchronization overhead, which becomes the primary factor affecting execution time in the parallel configuration. The spike in the parallel version, shown in Figure 5c, indicates the additional time taken for scattering operations. While the objective function remains the most time-consuming task, as in the sequential version, scattering and gathering times add to the total computation time. In the parallel implementation, perturbations are scattered across multiple processors, and rewards are gathered, contributing to the initial spike in the first loop as memory is assigned to each scattered perturbation (Figure 5e). The fluctuations seen in the remaining parts of Figure 5c are attributed to the gathering time required in the parallel version, with total times for each major task displayed in Figure 5e.

Figure 6a compares the predicted execution time for 100 iterations, calculated using Equation (10) with the parameters α=0.00018, L=0.0022, $\beta_{s}=1\times 10^{-9}$, and $\beta_{d}=9.46\times 10^{-5}$, against the actual time taken by the program. These parameters were determined by fitting the predicted curve to the actual data. The figure presents performance across different processor configurations (64, 128, 256, 512, and 1024 processors) within a single plot, illustrating the model’s accuracy. Therefore, Equation (12) effectively predicts time performance for various processor and worker configurations. From Equation (12), it can be observed that the optimal number of processors *p* depends on several key factors, including the number of workers $n_{w}$, the number of elements $n_{e}$, the communication startup cost *L*, and the computation cost per operation per element α. These parameters influence the scalability of the parallel algorithm. With $n_{w}=3600$, $n_{e}=2500$, L=0.0022, and α=0.00018, the optimal number of processors is approximately 42,903. This calculation illustrates how the algorithm efficiently scales with increasing *p*, even when handling larger workloads. Using Open_MPI in Python with the mpi4py library [36,37,38,39,40], linear speedup was achieved, as demonstrated in Figure 6c. This result highlights the capability of the parallel implementation of the Open_MPI algorithm to effectively scale by increasing the number of processors. Figure 6b demonstrates how varying the number of workers influences convergence speed. While the number of workers varied at 1000, 10,000, and 100,000, the number of processors was maintained at p=1000. At the 33rd iteration with 1000 workers, the 21st iteration with 10,000 workers, and the 8th iteration with 100,000 workers, it was found that the evolution strategy converged. This indicates that convergence is noticeably accelerated when the number of workers rises.

## 5. Conclusions

In conclusion, this paper proposes a parallel ES algorithm, a parallelized version of the sequential ES for AI-driven inverse design of multifunctional nanosurfaces. The proposed parallel ES achieved favorable speedup, reducing the computation time hundredfold when adequate computational resources are available; for instance, reducing the runtime from 5.6 days to 853.5 s. The comparisons between the nanopillar distribution, UV dose requirements, and cross-sectional surface profiles between the parallel and sequential ES show that both versions achieved a similar level of accuracy with color, friction, and bactericidal effectiveness. This scalable AI-driven inverse-design tool holds a notable potential for applications in broad nanomanufacturing and nanoscience areas. 

## Figures and Tables

**Table 1:** Sequential ES algorithm for multifunctional nanosurface development.

Algorithm: Sequential ES
**Input:** *n*, $n_{w}$, σ, and α.
**Output:** $\theta_{p}$, $r_{a}$, and G
1. Initialize parameter vector $\theta \in R^{3}$ with 100 // Initial guess values
2. Initialize $r_{a}\in R^{n}$ with 0 // Average reward
3. Initialize $r_{p}\leftarrow 0$ // Previous reward
4. Initialize r←0
5. Initialize $\theta_{p}\in R^{3}$ with 0
6. **For** c=1 to *n* **do**:
6.1 $E\leftarrow \left[E_{ij}∼N\left(0,1\right):i=1,2,\ldots ,n_{w};j=1,2,3\right]\in R^{n_{w}\times 3}$
6.2 Initialize $r\in R^{n_{w}}$ with 0
6.3 **For** w=1 to $n_{w}$ **do**:
6.3.1 $\epsilon_{w}\leftarrow \left\{E_{wj}:j=1,2,3\right\}\in R^{3}$
6.3.2 $\theta_{p}\leftarrow \theta +\sigma \epsilon_{w}$
6.3.3 **If** $\theta_{p_{k}}\leq 0$ for k∈{1,2,3}:
6.3.3.1 $r_{w}\leftarrow r_{p}$
6.3.4 **Else**:
6.3.4.1 $r,G\leftarrow \mathrm{Obj}(\theta_{p})$ // Objective function
6.3.4.2 $r_{w}\leftarrow r$
6.3.5 **If** r>0.96:
6.3.5.1 $r_{a_{c}}\leftarrow r$
6.3.5.2 **Return** $\theta_{p},r_{a},G$
6.4 $r_{a_{c}}\leftarrow E\left(r\right)$
6.5 $r_{p}\leftarrow r_{a_{c}}$
6.6 $w\leftarrow E^{T}\cdot r$
6.7 $\theta \leftarrow \theta +\frac{\alpha }{n_{w}\sigma }w$
7. **End for**

The Sequential ES algorithm steps involve calculating and updating parameters iteratively for multifunctional nanosurface development.

**Table 2:** Parallel ES algorithm for multifunctional nanosurface development using Open_MPI().

Algorithm: Parallel ES
**Input:** *n*, $n_{w}$, σ, and α
**Output:** $r_{a},\theta_{p},G_{w}$
1. Initialize MPI environment:
1.1 comm←MPI.COMM_WORLD
1.2 rank←comm.Get_rank()
1.3 size←comm.Get_size()
2. **If** $n_{w}<\mathrm{size}$:
2.1 $n_{w}\leftarrow \mathrm{size}$
3. Initialize parameter vector $\theta \in R^{3}$ with 100 // Initial guess values
4. Initialize $r_{a}\in R^{n}$ with 0 // Average reward
5. Initialize $r_{p}\leftarrow 0$
6. Initialize r←0
7. Initialize $\theta_{p}\in R^{3}$ with 0
8. **For** c=1 to *n* **do**:
8.1 **If** rank=0 **then**:
8.1.1 $E\leftarrow \left[E_{ij}∼N\left(0,1\right):i=1,2,\ldots ,n_{w};j=1,2,3\right]\in R^{n_{w}\times 3}$
8.1.2 $C_{1}^{\mathrm{size}}\leftarrow \left\{C_{k}=\left[E_{ij}:i=k+p\cdot \mathrm{size};\;j=1,2,3\right]:k=1,2,\cdots ,\mathrm{size}\right\}$
8.1.3 Initialize $r\in R^{n_{w}}$ with 0
8.2 **Scatter**: $\mathrm{comm}.\mathrm{scatter}\left(C_{k}\right)\forall k\in \left\{1,2,\cdots ,\mathrm{size}\right\}$
8.3 **Broadcast**: $\mathrm{comm}.\mathrm{bcast}(\theta ,r_{p},r)$
8.4 Initialize empty list:
8.4.1 $r_{w}\in R^{\mathrm{nrows}(C_{k})}$ with 0
8.5 **For** w=1 to $\mathrm{nrows}(C_{k})$ **do**:
8.5.1 $\epsilon_{w}\leftarrow \left\{{\left[C_{k}\right]}_{wj}:j=1,2,3\right\}\in R^{3}$
8.5.2 $\theta_{p}\leftarrow \theta +\sigma \epsilon_{w}$, where $\theta_{p}\in R^{3}$
8.5.3 **If** $\theta_{p_{k}}\leq 0$ for k∈{1,2,3}:
8.5.3.1 $r_{w}\leftarrow r_{p}$
8.5.4 **Else**:
8.5.4.1 $r_{w_{w}},G_{w}\leftarrow \mathrm{Obj}\left(\theta_{p}\right)$ // Objective function
8.5.4.2 **If** $r_{w_{w}}>0.96$:
8.5.4.2.1 $r_{a_{c}}$ ← $r_{w_{w}}$
8.5.4.2.2 **Return**: $r_{a},\theta_{p},G_{w},r_{w}$
8.6 **If** rank=0 **then**:
8.6.1 **Gather**:
8.6.1.1 $r_{f}\leftarrow {\left[r_{w}^{1^{⊤}},r_{w}^{2^{⊤}},\ldots ,r_{w}^{{\mathrm{size}}^{⊤}}\right]}^{⊤}\in R^{n_{w}}$
8.6.2 $r_{a_{c}}\leftarrow E\left(r_{f}\right)$
8.6.3 $r_{p}\leftarrow r_{a_{c}}$
8.6.4 $w\leftarrow E^{T}\cdot r_{f}$
8.6.5 $\theta \leftarrow \theta +\frac{\alpha }{n_{w}\sigma }w$
9. **End for**

The Parallel ES algorithm steps involve distributed parameter updates using MPI for multifunctional nanosurface development.

## Data Availability

Data will be made available upon request.
